# Structure-guided Discovery of Dual-recognition Chemibodies

**DOI:** 10.1038/s41598-018-25848-0

**Published:** 2018-05-15

**Authors:** Alan C. Cheng, Elizabeth M. Doherty, Sheree Johnstone, Erin F. DiMauro, Jennifer Dao, Abhinav Luthra, Jay Ye, Jie Tang, Thomas Nixey, Xiaoshan Min, Philip Tagari, Les P. Miranda, Zhulun Wang

**Affiliations:** 1 Department of Therapeutic Discovery, Amgen Discovery Research, Amgen Inc., 1120 Veterans Boulevard, South San Francisco, CA 94080 USA; 2 Department of Therapeutic Discovery, Amgen Discovery Research, Amgen Inc., One Amgen Center Drive, Thousand Oaks, CA 91320 USA; 3 Department of Therapeutic Discovery, Amgen Discovery Research, Amgen Inc., 360 Binney Street, Cambridge, MA 02142 USA

## Abstract

Small molecules and antibodies each have advantages and limitations as therapeutics. Here, we present for the first time to our knowledge, the structure-guided design of “chemibodies” as small molecule-antibody hybrids that offer dual recognition of a single target by both a small molecule and an antibody, using DPP-IV enzyme as a proof of concept study. Biochemical characterization demonstrates that the chemibodies present superior DPP-IV inhibition compared to either small molecule or antibody component alone. We validated our design by successfully solving a co-crystal structure of a chemibody in complex with DPP-IV, confirming specific binding of the small molecule portion at the interior catalytic site and the Fab portion at the protein surface. The discovery of chemibodies presents considerable potential for novel therapeutics that harness the power of both small molecule and antibody modalities to achieve superior specificity, potency, and pharmacokinetic properties.

## Introduction

Human antibodies are highly effective at binding protein surfaces, but have less success when a deep, concave pocket is the desired site of drug action^1^. On the other hand, small molecules have traditionally been used in targeting deep, concave pockets. However, over 80% of protein targets are thought to be intractable to small molecules with drug-like properties^2–4^. Here, we report an approach leveraging structure-guided design in the discovery of novel, small molecule-antibody hybrids, which we term “chemibodies,” to address therapeutic targets that are poorly amenable to small molecule or biologics approaches alone. These unique molecular hybrids have potential not only as novel therapeutics for difficult cell-surface targets such as ion channels, receptors, and membrane-associated enzymes, but also as valuable tools for elucidating the underlying biology of drug targets.

Conceptually, we leverage both small molecule and antibody modalities to synergistically target the same protein. Recently, a notable but rare subset of cow antibodies have been found to reach into deep protein pockets through unusual, ultra-long CDR H3 loops of around 60 amino acids^1^. Such long CDR H3 loops have not yet been reported for human antibodies. However, chemibodies can be thought of as mimics of these unusual cow antibodies, with additional precision and flexibility enabled by synthetic chemistry.

There is precedence for small molecule-antibody hybrids. The vast majority of work has been done with antibody drug conjugates (ADCs), where a non-specific small-molecule cytotoxin is tethered to a cell-specific antibody through a cleavable linker so that the toxin is released when the antibody is internalized into the cell through endocytosis^5,6^. In addition to ADCs using cleavable linkers, there are several recent reports where antibodies against tumor antigens were tethered through a non-cleavable linker to a small molecule that can impart cytotoxicity by binding to CD3, Na,K-ATPase, tublin or cell surface antigen, such as Kadcyla which is an ADC consisting of the cytotoxic agent emtansine DM1 directly attached to trastuzumab (Herceptin) and approved for treatment of HER2-postive metastatic breast cancer^7–9^.

Instead of cytotoxicity, we seek superior modulation of the targets’ activity with properties conducive to becoming a human therapeutic for treating grievous illnesses. Our approach uses high-resolution structural data to rationally design small molecule-antibody hybrids that are capable of dual recognition of both a surface epitope and a deep, buried substrate catalytic site of the same enzyme. Since we wish to target the region in the antibody closest to the catalytic site, the small molecule conjugation point on the antibody may not be in the constant region as with ADCs. The ability to precisely attach and orient components of the small molecule-antibody hybrids becomes critical for providing the synergistic potency and selectivity we seek in chemibodies.

In the current study, we embarked on a structure-guided engineering effort to discover a superior inhibitor for dipeptidyl peptidase IV (DPP-IV) enzyme as a proof of concept study. DPP-IV is a target for several marketed small molecule inhibitors for the treatment of type II diabetes, including linagliptin, sitagliptin, vildagliptin, saxagliptin, and alogliptin^10^. Since the available small molecule drugs have limitations in terms of efficacy and side effects^10^, we sought an alternative strategy for inhibiting DPP-IV activity.

We previously reported discovery of a panel of mouse monoclonal antibodies (mAbs) against rat DPP-IV that could complement known competitive small molecule inhibitors^11^. These inhibitory antibodies to rat DPP-IV showed notable *in vivo* efficacy in hyperglycemic Zucker fatty rats in terms of improving glucose tolerance. The tightest binding antibody, 11A19, had a measured K_d_ of 10 pM for rat DPP-IV. However, the IC_50_ for DPP-IV inhibition was 0.8 nM for the GLP-1 peptide substrate, with only partial inhibition; the enzyme activity was reduced by only 60–80% at saturating antibody concentrations.

To understand the molecular mechanism of the partial inhibition of the 11A19 antibody, we previously solved co-crystal structures of 11A19 Fab with DPP-IV catalytic domain (PDB code: 4FFV), which showed that the Fab does not access the catalytic site, but instead partially blocks the “side” opening, which is believed to be one of the entries to the catalytic site^11^. Interestingly, the structural data also revealed a possibility for small molecules to access the active site in the presence of the blocking antibody, 11A19. Indeed, we were able to generate a co-crystal structure of the ternary complex of rat DPP-IV with both 11A19 Fab and a small molecule inhibitor sitagliptin (PDB code: 4FFW), demonstrating the simultaneous binding of the two molecules, with the Fab on the surface and the small molecule inhibitor in the deep catalytic pocket^11^. Together, the two structures rationalize the partial inhibitory activity profile of the antibodies towards peptide substrates due to the fact that the antibody was not fully blocking the active site.

Moreover, the ternary structure revealed an attractive opportunity to link 11A19 Fab and sitagliptin together, and inspired us to apply a rational design and synthetic chemistry approach to engineering a hybrid. We hypothesized that such a hybrid could be more potent than either the antibody or small molecule alone due to the chelate effect^12^, and that the small molecule would confer improved inhibition of DPP-IV as well as improved selectivity.

## Results

**Figure 1:**
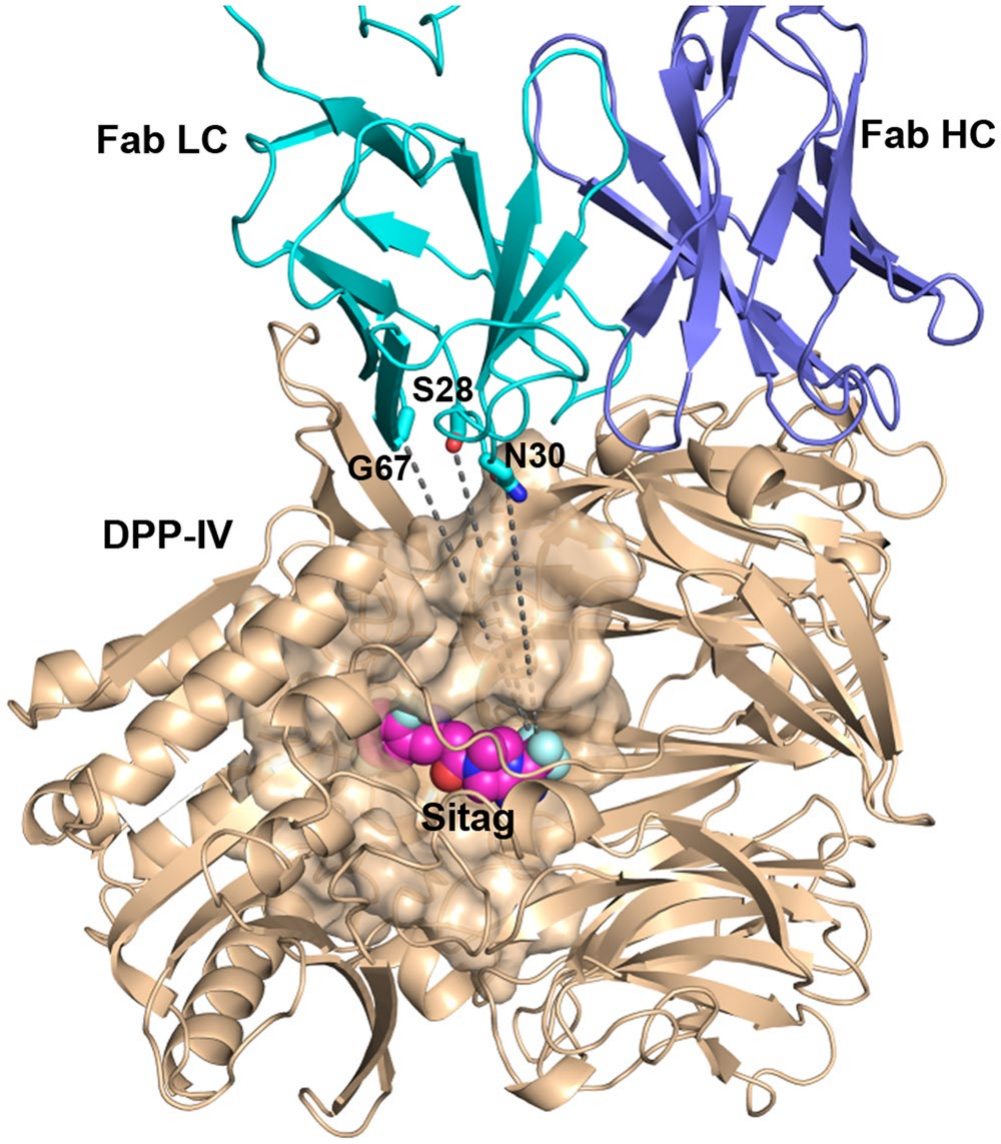
Co-crystal structure of the ternary complex of DPP-IV (wheat ribbons and surface) bound with 11A19 Fab (teal and blue ribbons) and small molecule (SM) **1** (spheres). PDB code: 4FFW. Three residues on the Fab identified as potential conjugation sites are labeled and shown in stick.

To generate a small molecule-antibody hybrid, we needed first to engineer antibody variants and small molecule analogs as precursors for conjugation reactions. On the antibody side, we looked to engineer a cysteine residue that would provide a reactive thiol handle for conjugation of a PEG linker. Using our ternary structure of DPP-IV complexed with 11A19 Fab and sitagliptin (PDB code: 4FFW), we looked for antibody residue positions that met four criteria: high solvent exposure, minimal involvement in DPP-IV recognition, short distance to the bound small molecule inhibitor, and side-chain oriented in the direction of the substrate pocket. We identified three suitable residues in the 11A19 light chain for mutation to cysteine residues: Ser28 and Asn30 of CDR L1, and Gly67 of the framework (Fig. 1). All three Cys variants of 11A19 were generated successfully. The K_d_ binding affinities of S28C, N30C, and G67C mutated Fab variants to DPP-IV were assessed by Biacore SPR to be 5.7, 2.9, and 11.0 nM, respectively (Supplementary Table S1). Compared with the wild type Fab, with a K_d_ of 1.8 nM, the N30C Fab variant had the least impact on binding, with less than a two-fold loss of K_d_ affinity.

**Figure 2:**
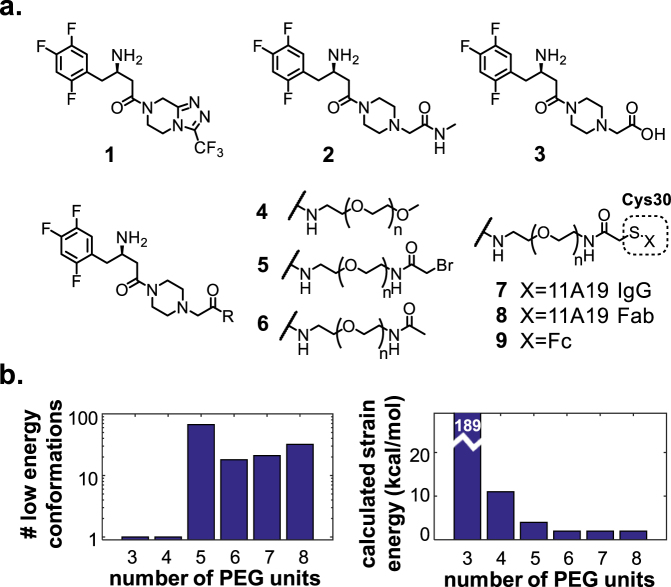
Designed small molecule (SM) analog structures. (**a**) Hybrid molecule design. For **4**–**9**, n indicates number of PEG units. (**b**) Computational modeling and energetic favorableness of **8** with 3–8 PEG units. Left panel: number of low energy conformations in the modeled bound conformation. Right panel: calculated strain energy in lowest energy conformation found, where lower strain energies are expected to be favorable. See Methods for additional details.

On the small molecule side, known structure-activity-relationships^13,14^ of DPP-IV inhibitors together with the inhibitor co-crystal structure informed design of small molecule analogs likely to largely retain potency while enabling conjugation chemistry. We started from sitagliptin, compound **1** (Fig. 2), designed and synthesized an amide analog of sitagliptin, compound  **2** (Figs 2 and S1). The activity of **2** was measured to have a potency of IC_50_ = 130 nM, with a limited potency loss of seven-fold as compared with that of sitagliptin which showed a potency of IC_50_ = 19 nM under the same experimental conditions. We then prepared the acid analog, **3**, to serve as an intermediate for conjugation. We did not further optimize **2**, but published data suggests that further optimization, particularly around the piperazine ring^14^, should improve potency. Addition of PEG_3_-OMe and PEG_7_-OMe to **3** gave analog **4** (with n = 3 and n = 7, respectively), which had moderately improved IC_50_’s of 105 nM and 70 nM, respectively (Fig. 2).

With the successful identification of suitable 11A19 variants, small molecule sitagliptin analogs, and tethering points, we next looked at designing a linker between the biologic and small molecule to span a distance of ~28 Å based on crystal structure observations (Fig. 1). To identify an optimal linker, we modeled the bound state of several linker compositions and gauged their flexibility and strain computationally by performing extensive conformational searches (see *Methods* for details). A linker which is too short to deliver a potency boost would have high strain. On the other hand, a linker that is too long would have a high number of thermodynamically-accessible linker conformations and would begin to lose the synergistic effects on potency^15^. In addition, different linker lengths have different conformational preferences. We modeled a number of peptide-based linkers and initially tested poly-Ala, but PEG had much improved solubility. Molecular modeling of the molecules followed by estimation of linker conformations and linker strain, as shown in Fig. 2b, suggested that five or six PEG units would be optimal to maintain the desired bivalent inhibitor binding mode and also to provide synergistic binding affinity.

To assemble the complete hybrid molecules, the engineered cysteine residues (Cys30) in the Fab and IgG variants were reduced with tris-(2-carboxyethyl)phosphine (TCEP). Acetylbromide-derivatized analogs of **4** with PEG linkers varying in length (**5** with n = 3–8) were then conjugated to the reduced residue N30C of 11A19 Fab or IgG variants (Figs 2 and S3). A series of analogs, **6**, with a PEG linker and no Fab or antibody attached, were synthesized as negative controls (Fig. 2). A series of chemibodies, **7**–**9**, were generated by conjugating analog **5** with 11A19 IgG and 11A19 Fab harboring N30C mutations, as well as to a negative control Fc molecule (Fig. 2), respectively. Mass spectrometry analysis was used to confirm the covalent attachment and calculate the ratio of small molecule inhibitor attached to Fab or IgG (Supplementary Tables S2–S5). The conjugated products were characterized by various methods, including RPLC/TOF-MS, SEC and SDS-PAGE. The purity of the products was >90% with more than 85% in the monomeric form. Additional details for the conjugation chemistry are described in the online Methods.

**Figure 3:**
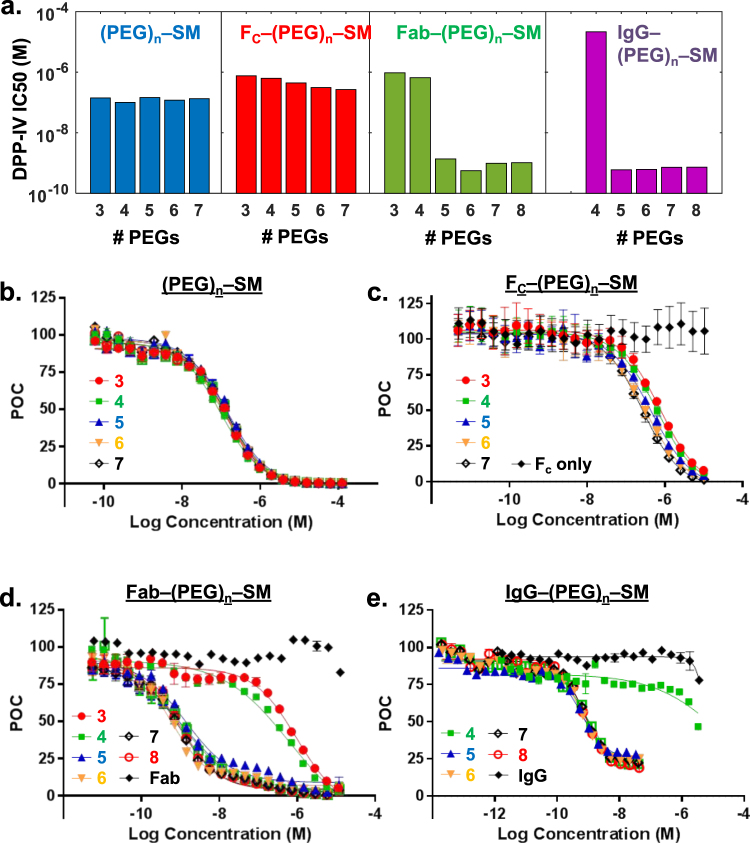
Biochemical assay results. (**a**) Measured DPP-IV IC50′s for small molecule (SM) inhibitor conjugated to PEG linkers with variable numbers of ethylene oxide units (**6** with n = 3–7), through PEG linkers to F_c_ (**9** with n = 3–7), through PEG linkers to N30C of 11A19 Fab (**8** with n = 3–8), and through PEG linkers to N30C of 11A19 mAb (**7** with n = 3–8). (**b**–**e**) IC_50_ curves corresponding to the molecules in panel (a). Y-axis label POC stands for Percent of control with 100% being cleavage of the dipeptide substrate with no inhibitor added and 0% being full inhibition. All measurements were done in triplicate and error bars are provided for (**b**–**e**). Note only some error bars are noticeable due to very tight assay results. Data for additional molecules with longer PEG linkers are provided in the Supplemental Materials.

Next we assessed the DPP-IV activity on dipeptide substrate cleavage with these hybrid chemibodies. IC_50_ results for these molecules are shown in Fig. 3 and Supplementary Table S6. Analogs **6**, with PEG linker but no Fab or antibody tethered, had similar potency to analog **2** irrespective of the number of PEG units in the linker. Analogs **8** (with Fab attached) showed strikingly distinct activity differences between molecules with 3–4 PEG units and molecules with 5–8 PEG units. The molecule with 6 PEG units and Fab had the lowest IC_50_ at 0.56 nM, a 212-fold increase in potency over the molecule without Fab (**4**, with n = 6). The 6-PEG full IgG hybrid (**7**, with n = 6) had a similar IC_50_ of 0.6 nM. The molecule **7** (n = 6) had a 32-fold increase in potency relative to sitagliptin, and is similar to or slightly more potent than the most potent inhibitor known, linagliptin, which has a reported IC_50_ of ~1 nM^16^.

In addition, this initial hybrid molecule has substantial room for further improvement. For instance, known SAR on the small-molecule portion suggests that analogs with a 2-benzyl on the piperazine ring can improve potency by 5–10 fold^14^. To further validate our results, we performed control experiments where molecules that were conjugated to a non-targeting Fc molecule through E384C showed IC_50_’s of 1–2 uM (Fig. 3).

**Figure 4:**
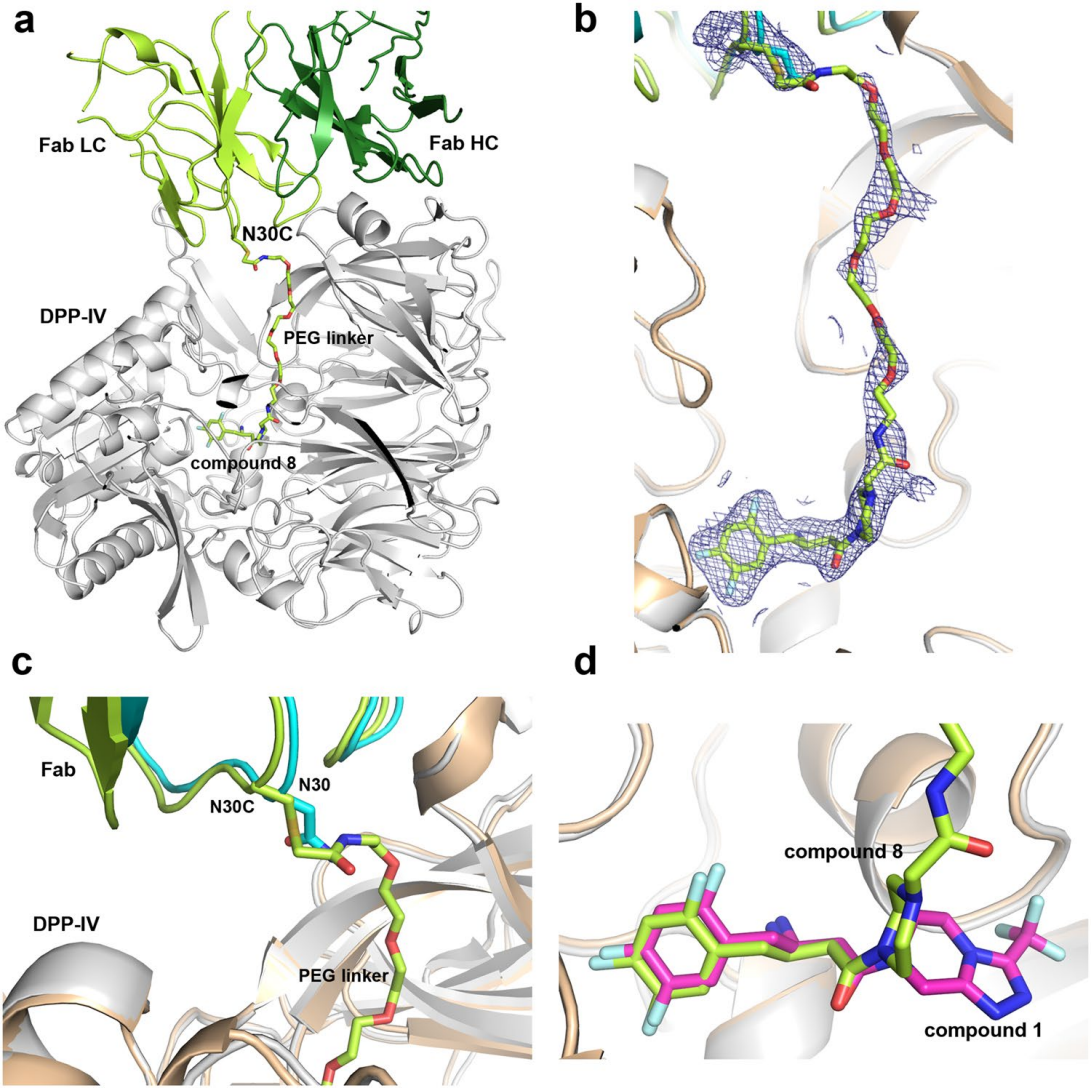
Co-crystal structure of DPP-IV complexed with a chemibody of small molecule-antibody hybrid 11A19 Fab-(PEG)6-SM (**8** with n = 6). (**a**) Overall structure with DPP-IV (grey ribbons), Fab light chain (lime green) and heavy chain (forest green), and chemically linked SM (green carbons, red oxygens, blue nitrogens, cyan fluorines). (**b**) Electron density of SM and linker. Blue mesh, 2fo-fc map contoured at 1.0 sigma. (**c**) Close-up of conjugation site at N30C of 11A19 Fab light chain, overlaid with the ternary complex structure (4FFW) of DPP-IV (beige) with 11A19 Fab (light chain in cyan). (**d**) Superposition of the SM portion of the hybrid (compound **8**) (green) and sitagliptin (compound **1**) (magenta).

To confirm and understand the molecular basis of the activities of these hybrid chemibodies, we solved a co-crystal structure of a 6-PEG Fab chemibody (**8**, n = 6) complexed with DPP-IV at 2.77 Å resolution (Fig. 4 and Supplementary Table S7). The structure reveals the simultaneous dual binding of both the small molecule inhibitor and the Fab, with the linker also being largely resolved (Fig. 4a,b). The CDR-L1 loop where residue Asn30 was engineered into Cys30 (Fig. 4c) shows no substantial conformational changes caused by the mutation and addition of the linker, as compared with that of wild type 11A19 Fab alone. In fact, Cys30 maintains the same rotamer conformation (phi, psi, Cα-Cβ dihedrals) as observed in the original Fab crystal structure with residue Asn30. On the small molecule inhibitor side, shown in Fig. 4d, comparison with sitagliptin shows that the trifluorophenyl ethylamine portion of the molecule is largely unperturbed. The piperidine adopts an alternate pucker due to both the modification of the compound as well as, likely, the influence of the PEG linker. As a result, the small molecule ligand portion has fewer interactions with the protein as compared with the parent molecule sitagliptin. The crystal structure provides not only an understanding of the successful structure-guided design of this small molecule-antibody hybrid, but also exceptionally valuable insight for further improvement of the chemibody targeting DPP-IV.

## Discussion

Using co-crystal structures of 11A19 bound to DPP-IV, and of sitagliptin bound to DPP-IV, we were able to rapidly identify modifications on both the antibody side and small molecule side to enable conjugation through a PEG linker. Computational analysis of linker strain helped identify a range of linkers that were likely to provide synergistic binding of antibody and small molecule at their respective sites. The measured IC_50_’s of the molecules after synthesis correlate well with the calculated strain energy, with Pearson correlations of 0.81 and 0.97 with the Fab and IgG chemibody IC_50_’s, respectively, suggesting that the computational strain calculations we applied are useful in the design of chemibody linkers. The experimental results show that linkers with three or four PEG units are too short to provide inhibition at the DPP-IV catalytic site, while linkers of five to eight PEG units result in similar IC50′s, with six PEG units providing the most favorable IC_50_. The flexibility of the PEG linker appears to allow correct binding of the small molecule with linkers of varying number of PEG units, as long as the linker is not too short. We expect that very long PEG-based linkers might have negative impact to inhibition since the limited size of the protein pocket would result in crowding of the linker. The computational strain calculations may be even more useful for more rigid, non-PEG linkers.

The 6-PEG Fab chemibody (**8**, n = 6) had a measured DPP-IV IC_50_ of 0.56 nM, a 212-fold increase in potency over the parent analog, **2**, and a 32-fold increase in potency over sitagliptin. The 6-PEG IgG chemibody (**7**, n = 6) had a nearly identical IC_50_ potency. Known structure-activity-relationships suggest that analoging of the small molecule component at the piperazine may yield an additional 5–10x increase in potency. Relative to a negative control, F_c_-(PEG)_6_-SM, the 6-PEG Fab and IgG chemibodies are over 500-fold more potent.

With respect to the antibody, 11A19, the chemibody results in potent inhibition of dipeptide cleavage by DPP-IV, where no detectable dipeptide cleavage inhibition was observed with antibody alone. This is consistent with the observation that 11A19 does not directly block the catalytic site, but instead partially blocks the “side” opening. The chemibodies not only partially block the substrate path but also occupy the catalytic site. We did not measure selectivity of the chemibody across the DPP family of enzymes, but inspection of the sequence alignment for DPP-IV at the antibody binding site finds the binding site to be unique, and thus the chemibodies are likely highly selective.

Recently, a notable but rare subset of cow antibodies have been found to reach into deep pockets through unusual, ultra-long CDR H3 loops. Such antibodies have not been reported thus far for human antibodies. However, one may imagine chemibodies as synthetic mimics of these unusual cow antibodies, with perhaps additional precision and flexibility enabled by synthetic chemistry.

In conclusion, we have described the structure-guided design and discovery of a chemibody (small molecule-antibody hybrid) targeting the same biological target at dual binding sites, with potential to preserve advantages of the large molecule, such as longer half-lives and exquisite selectivity, while imparting superior inhibitory activity. We believe this approach can be useful for many targets, including surface-expressed enzymes, ion channels, and transporters. We demonstrate the critical enabling role of high-resolution structural data in our ability to precisely and rationally design chemibodies with dual targeting activity. Conceptually, an antibody can improve the selectivity, potency, kinetics, and half-life of a non-selective or insufficiently potent small molecule modulator. Correspondingly, a small molecule can access catalytic sites or active sites that are often inaccessible to antibodies. The additional potency and selectivity boost from an antibody component can be valuable for binding pockets that are difficult to target with a small molecule alone. In addition, the chemibodies we presented here can be either in Fab-conjugate or IgG-conjugate formats. While both formats grant the same potency and selectivity, IgG-conjugates provide important pharmacokinetics and effector function endowed by the Fc region. Fab-conjugates, however, have values in more efficient penetration of tissue selection with lower molecular weight (100 kDa less than IgG) and elimination of Fc-associated effector functions as well as facilitating structural analysis. Finally, while the work here demonstrates chemibodies as inhibitors, the approach we have taken could potentially be pursued for activators as well.

## Methods

### Computational modeling of molecules

Fab-conjugated molecules with PEG linkers with 3–8 ethylene oxide units were modeled based on our previously reported co-crystal structure (PDB ID: 4FFW), and evaluated using extensive conformational searches with the antibody Fab and the active site small molecule fixed in place. The results of the searches were used to calculate two quantitative measures of how favorable the linker would be energetically upon binding to DPP-IV. The first measure is the number of low energy conformations, defined here as the number of conformations calculated to be within 2.0 kcal/mol of calculated ground state, and results are shown in the left panel of Fig. 2b. Higher numbers of conformations are expected to be favorable because of a lesser entropic penalty upon binding to DPP-IV, while a lower number of conformations are expected to contribute to synergistic binding. The minimum number of conformations is one because there must be at least one ground state. The second measure is the strain energy in the lowest energy conformation found, where lower strain energies are expected to be favorable. Results are shown in the right panel of Fig. 2b. The PEG_3_ molecule has a calculated strain of 128 kcal/mol due to the linker being too short to connect the desired bound position of the competitive small molecule inhibitor and Cys30 in 11A19 Fab. Searches were done with low-mode MD conformational search in MOE 2012 (Chemical Computing Group, Montreal) with the MMFF94x force-field, RMSD cutoff = 0.25 A, and Reject Limit = 300. The small molecule up to and including the piperazine ring was modeled based on a co-crystal structure (PDB code: 4FFW) and fixed in place during the conformational search.

### Preparation of IgG and Fab

mAb variants were expressed and purified as previously described^11^. Briefly, mAbs were expressed in 293 6E cells and captured on MabSelect SuRe columns, which had been equilibrated with HEPES buffered saline solution HBS (25 mM HEPES, 150 mM NaCl, pH 7.6). After washing with HBS, bound IgG proteins were eluted with 0.5% acetic acid, pH 3.5. Eluted IgG molecules were further polished on SP–Sepharose or Superdex 200 to remove aggregates in HBS. The Fab fragment was prepared by digesting full antibody molecules with immobilized papain (Thermo Scientific) according to the manufacturer’s instructions. The Fab molecules were recovered by passing through a MabSelect SuRe column for Fc fragment removal.

### Synthesis of DPP-IV inhibitor, (R)-2-(4-(3-amino-4-(2, 4, 5-trifluorophenyl) butanoyl) piperazin-1-yl)-N-methylacetamide hydrochloride (compound **2**)

The synthetic scheme is depicted in Supplementary Fig. S1. We prepared **10** using the synthesis reported by Kim *et al*.^13^. To a solution of compound **10** (800 mg, 2.4 mmol) and N-methyl-2-(piperazin-1-yl)acetamide (376 mg, 2.4 mmol) in dimethylformamide (20 mL), NEt_3_ (1.04 mL,7.2 mmol) and propyl phosphonic anhydride (50% in ethyl acetate, 2.2 mL,7.2 mmol) were added at 0 °C and the reaction mixture was allowed to stir at 25 °C for 16 h. Water (50 mL) was added to the reaction mixture and extracted with ethyl acetate (50 mL × 2). The organic layer was dried over Na_2_SO_4_, filtered and concentrated to obtain the crude intermediate which was further purified by column chromatography using silica gel (100–200 mesh) and 0–5% methanol in dichloromethane to obtain 300 mg (27%) of intermediate amide **10** as an off white solid. MS (ESI, positive ion) m/z: 473.3 [M + 1]. Amide **10** (300 mg, 0.63 mmol) was treated with 4 N HCl in dioxane (10 mL) at 0 °C and the reaction mixture was allowed to stir at 25 °C for 2 h. The reaction mixture was concentrated to a solid that was washed with diethyl ether to obtain 150 mg (64%) of the HCl salt of compound **2** as an off-white solid^1^.H NMR (400 MHz, DMSO) δ 7.73 (s, 1 H), 7.44 (dt, *J* = 17.8, 6.9 Hz, 2 H), 3.50–3.41 (m, 4 H), 3.26–3.20 (m, 1 H), 2.90 (s, 2 H), 2.67 (dd, *J* = 13.1, 5.7 Hz, 1 H), 2.61 (d, *J* = 4.7 Hz, 3 H), 2.59–2.53 (m, 1 H), 2.42–2.37 (m, 2 H), 2.33 (t, *J* = 6.6 Hz, 4 H). MS (ESI, positive ion) *m/z*: 373.15 [M + 1].

### Preparation of conjugating reagents and PEGylated control acetamides

#### (R)-ethyl 2-(4-(3-((tert-butoxycarbonyl) amino)-4-(2, 4, 5-trifluorophenyl) butanoyl) piperazin-1-yl) acetate

Synthetic scheme is depicted in Supplementary Fig. S2. To a solution of compound **10** (11.0 g, 33 mmol) and ethyl 2-(piperazin-1-yl)acetate (5.68 g 33 mmol) in dimethyl formamide (100 mL), NEt_3_ (11.8 mL, 82.5 mmol) and propyl phosphonic anhydride (T_3_P, 50% in ethyl acetate) (52.4 mL, 82.5 mmol) were added at 0 °C and the reaction was allowed to stir at 25 °C for 16 h. Water (500 mL) was added to the reaction mixture and extracted with ethyl acetate (500 mL × 2). The organic part was dried over Na_2_SO_4,_ filtered and concentrated to obtain the crude which was further purified by column chromatography using silica gel (100–200 mesh) and 0–5% methanol in dichloromethane to obtain 10 g (62%) of the intermediate amide as an off-white solid. MS (ESI, positive ion) m/z: 311.7 (M + 1)^1^.H NMR (DMSO-*d*_6_, 400 MHz) δ ppm 1.27(t, *J* = 7.2 Hz, 3 H), 1.35(s,9 H), 2.48–2.60(m, 6 H), 2.88–2.95(m, 2 H), 3.23(s, 2 H), 3.41–3.45(m, 2 H), 3.63–3.72(m, 2 H), 4.07–4.10(m, 1 H), 4.18(q, *J* = 7.0,2 H), 5.61(d, *J* = 8.8, 1 H), 6.88(q, *J* = 8.5, 1 H),7.06(q, *J* = 8.4, 1 H).

#### (R)-2-(4-(3-((tert-butoxycarbonyl) amino)-4-(2, 4, 5-trifluorophenyl) butanoyl) piperazin-1-yl) acetic acid

To the intermediate above (9 g, 18.4 mmol) in THF: MeOH: H_2_O (5:3:1) (90 mL), LiOH:H_2_O (1.54 g, 36.5 mmol) was added at 0 °C and reaction mixture was allowed to stir at 25 °C for 2 h. The reaction mixture was concentrated; water (100 mL) was added and washed with ethyl acetate (100 mL × 2). The aqueous part was acidified with citric acid and extracted with 10% methanol in dichloromethane (200 mL × 3). The organic parts were dried over Na_2_SO_4,_ filtered and concentrated to obtain 5 g (59%) of compound **11** (Supplementary Fig. S2) as an off-white solid^1^.H NMR (400 MHz, DMSO) δ 7.45 (dd, *J* = 16.9, 10.3 Hz, 1 H), 7.29 (d, *J* = 9.6 Hz, 1 H), 6.73 (d, *J* = 9.1 Hz, 1 H), 4.01 (br s, 1 H), 3.59–3.27 (m, 5 H), 3.14(s, 2 H), 2.84 (d, *J* = 13.6 Hz, 1 H), 2.64–2.35 (m, 5 H), 1.27 (s, 9 H); 460.26(M + 1).

In a screw-cap vial, a solution of acid **11** (75 mg, 0.16 mmol) at 0.2 M in DMF was treated with HATU (1.1 eq) followed by azido-PEG-amine (1.2 eq) and finally diisopropylethyl amine (3 eq). The reaction mixture was agitated for 4 hr at ambient temperature. The reaction mixture was diluted in 10 mL EtOAc, washed with 1 N HCl (5 mL), satd NaHCO3 (5 mL), satd NaCl (5 mL), dried over MgSO4, filtered through a sintered glass funnel and concentrated *in vacuo* to afford azido intermediates **12a**–**h** as off-white residues.

The crude product was dissolved in 10% water in THF (1.6 mL) and treated with ~5 eq triphenyl phospine on PS resin (400 mg, loading 2.06 mmol/g, PS-triphenylphosphine, Biotage). The suspension was incubated at 80 °C for 1 h, then allowed to cool to room temperature and filtered to remove resin. The filtrate was concentrated *in vacuo*. To a screw cap vial was added PS-Carbodiimide PS-CDI 0.88 mmol/g (1.455 g, 1.280 mmol) followed by DCM (25 mL). The suspension was treated with 2-bromoacetic acid (0.222 g, 1.600 mmol) and shaken for 10 min. The crude amine was added as a solution in 5 mL DCM. The suspension was shaken at ambient temperature for 3 h, LC/MS of crude showed complete reaction. The suspension was filtered and the resin washed with DCM and MeOH. The combined filtrate was concentrated *in vacuo* to afford the crude product. The crude Boc-protected product was treated with 5 mL 1:1 DCM:TFA with 1% TIS and 1% water and stirred for 30 min at ambient temperature. The solution was concentrated *in vacuo*, azeotroping with several portions of DCM to afford the crude product as a TFA salt.

The product was purified by reverse-phase preparative HPLC: Phenomenex Gemini column, 10 micron, C_18_, 100 Å, 150 × 30 mm, 0.1% TFA in CH_3_CN/H_2_O at a flow rate of 60 ml/min; gradient − 10% to 40% over 15 minutes. Fractions containing product were pooled and lyophilized in a tared tube to afford the product as a sticky white residue, >95% purity by LC at 254 nm for the acetamides **6a–h**; >80% purity by LC at 254 nm for the bromoacetamides **5a–h** (minor product was the result of hydrolysis of the bromide to an unreactive hydroxyacetamide) (Supplementary Fig. S2). For assay, the acetamides **6a–h** were re-dissolved in DMSO at a concentration of 10 mM. For the conjugations, solutions of the bromoacetamides **5a–h** were prepared in DMF at either 1.7 mM or 6.8 mM (Supplementary Fig. S2; Tables S2 and S3).

### Conjugation of DPP-IV inhibitors to engineered N30C 11A19 Fab and IgG

#### IgG conjugates **7a**–**f**

The 11A19 IgG N30C mutant (2 mg) was treated with 6 molar equivalents of tris-(2-carboxyethyl) phosphine (TCEP, 4 mM) at ~1 mg/mL in pH 7.6 HEPES buffer and incubated for 40 min. The TCEP was removed by desalting into pH 7.6 buffer using a Zeba 40 MWCO desalt cartridge. The reduced IgG was treated with 9 molar equivalents of dehydroascorbic acid (DHAA, 4 mM) and incubated for 30 min followed by treatment with 12 molar equivalents of bromoacetamide (1.7 mM in DMF). The reaction mixture was allowed to incubate for 16 h, then desalted into pH 7.6 HEPES buffer and concentrated to ~300 uL using an Amicon 10 K MWCO centrifugal filter. The crude conjugate was purified by SE-HPLC, method attached, and fractions containing product combined and dialyzed into assay buffer (50 mM Tris, 100 mM NaCl, pH 7.8). The conjugate solution was concentrated using an Amicon 3 K MWCO filter and characterized by UV, gel electrophoresis, SEC, and RPLC/TOF-MS (Supplementary Figs S3 and S5; Table S5).

#### Fab conjugates **8a**–**h**

The 11A19 Fab N30C mutant (500 ug) was treated with 1.5 molar equivalents of tris-(2-carboxyethyl) phosphine (TCEP, 0.8 mM) at ~2.5 mg/mL in pH 7.6 50 mM HEPES, 150 mM NaCl buffer and incubated for 60 min at 20 °C. The reduced Fab was treated with 5 molar equivalents of bromoacetamide (6.8 mM in DMF). The reaction mixture was allowed to incubate for 5 h at 20 °C, then stood overnight at 4 °C, and finally desalted into assay buffer (50 mM Tris, 100 mM NaCl, pH 7.8) using a Thermo Pierce 10 K MWCO Zeba desalt 96-well plate (Supplementary Fig. S3). The final desalt step was repeated before the crude conjugate was characterized by UV, gel electrophoresis, SEC, and RPLC/TOF-MS (Supplementary Figs S3 and S4; Table S4).

### Conjugation to engineered E384C Fc control

The Fc conjugation reactions were performed similarly to that described for Fab conjugation, except reactions were performed in 50 mM sodium phosphate plus 2 mM EDTA pH 7.5 buffer, instead of 50 mM HEPES plus 150 mM NaCl pH 7.6 buffer.

### Determination of DPP-IV catalytic activity

DPP-IV catalytic activity and inhibition of dipeptide cleavage was measured using a fluorescent assay performed at room temperature in 384-well, black polypropylene microplates. 0.125 nM DPP-IV enzyme was mixed with 0–128 µM of Ala-Pro-AFC (Calbiochem 125510) in Reaction Buffer (50 mM Tris (pH 7.8), 100 mM NaCl, 0.01% (w/v) BSA, 0.01% (w/v) CHAPS and 1 mM DTT) containing varying amounts of inhibitor. The reaction was monitored continuously on the Infinite M1000 (Tecan) at 400 nm Ex/505 nm Em and the resulting data was analyzed using non-linear regression of Michaelis-Menton kinetic parameters. Inhibitor IC_50_ measurements were performed in a similar format in triplicates or more using Ala-Pro-AFC at 10 µM. Assay data shown in Fig. 3 were performed with a one-hour incubation time. The data were fit to the four-parameter model using a Levenburg–Marquardt nonlinear regression algorithm. Figure 3b,c were generated using GraphPad Prism v6.

### Protein reagents and crystallography

Rat DPP-IV enzyme and Fab mutant proteins were expressed and purified as previously described^11^. The purified DPP-IV/Fab 11A19-sitagliptin hybrid complex was concentrated to 6.6 mg/ml in buffer containing 20 mM Tris 7.9 and 200 mM NaCl. Crystals were grown at 20 °C in sitting drop trays with 0.1 µl protein solution plus 0.1 µl reservoir solution of 22% PEG 1000 and 0.1 M sodium citrate tribasic dihydrate pH 5.5. For x-ray diffraction data collection, crystals were transferred into well solution with 30% (w/v) ethylene glycol and then flash frozen in liquid nitrogen.

X-ray diffraction data sets were collected at beamline 502 at Advanced Light Source (ALS) (Berkeley, CA) and processed with programs MOSFLM^17^ and SCALA^18^ in the CCP4 program suite^19^. The structure was solved to a resolution of 2.77 Å by the molecular replacement program Phaser^20^ using a previously reported complex structure of rat DPP-IV with Fab 11A19 (PDB code: 4FFV) as a search model. Model building and refinement were carried out in COOT^21^ and REFMAC^22^ in CCP4, respectively. All structural figures were prepared using PyMOL (Schrodinger Inc, San Diego, CA).

### Accession code

The atomic coordinates and structural factors of DPP-IV and 11A19 Fab-(PEG)_6_-inhibitor complex have been deposited in the Protein Data Bank under accession code 5VTA.

## Electronic supplementary material

Supplementary Information
