# Supplementary material for: Structure-guided Discovery of Dual-recognition Chemibodies
Source: Sci Rep. 2018 May 15;8:7570. doi: 10.1038/s41598-018-25848-0 (PMC5954141; doi:10.1038/s41598-018-25848-0)
Supplement: Supplementary file 1 — Supplementary Information [file 41598_2018_25848_MOESM1_ESM.pdf]

Structure-guided Discovery of Dual-recognition Chemibodies

Alan C. Cheng[‡], Elizabeth M. Doherty[‡], Sheree Johnstone[‡], Erin F. DiMauro[‡], Jennifer Dao[‡], Abhinav Luthra, Jay Ye, Jie Tang, Thomas Nixey, Xiaoshan Min, Philip Tagari, Les P. Miranda* and Zhulun Wang*

Supplementary information

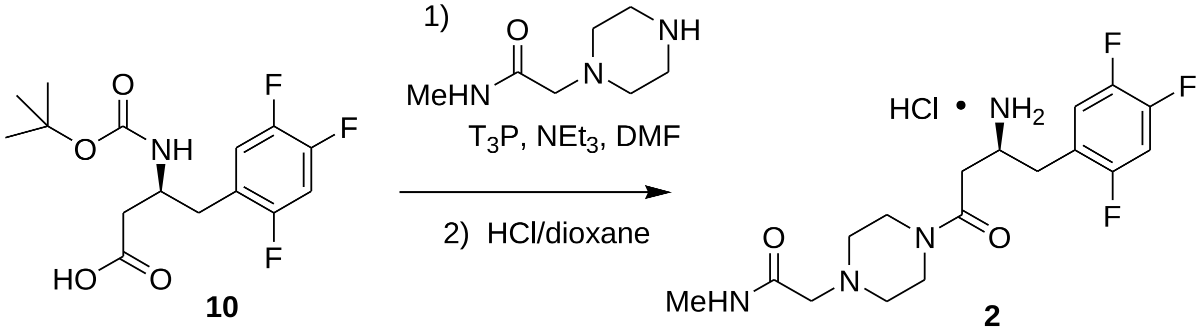

Figure S1. Synthesis of compound **2**.

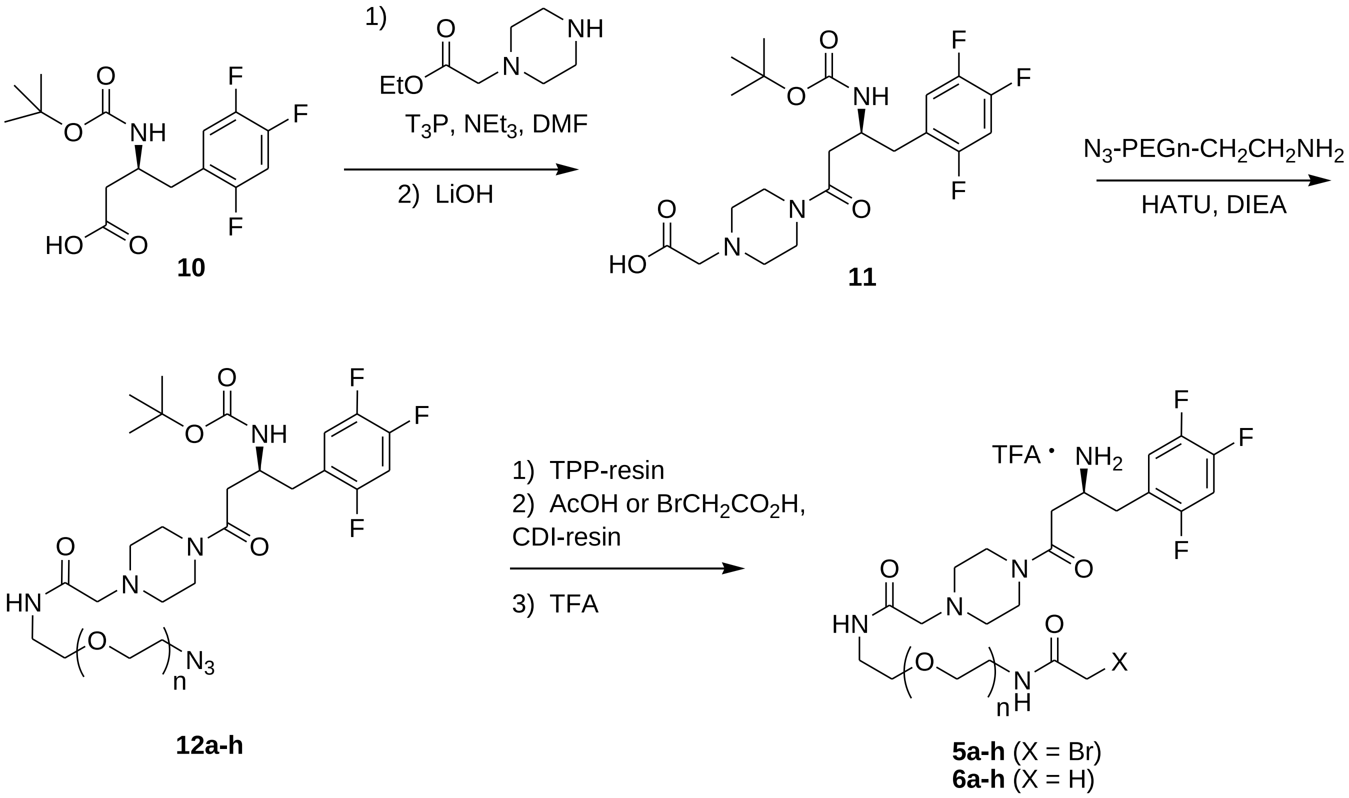

Figure S2. Preparation of conjugating reagents and PEGylated control acetamides.

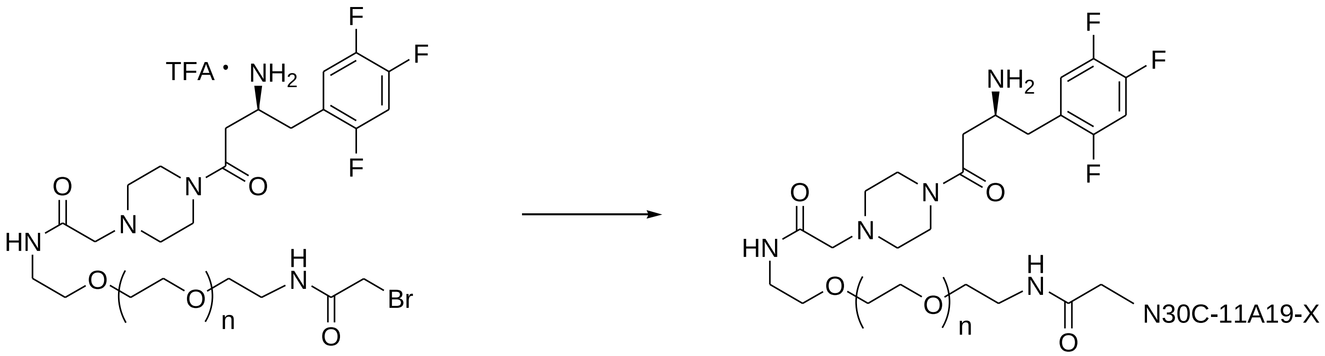

5a-h

7a-f (X=IgG)
8a-h (X=Fab)

Figure S3. Conjugation of DPP-IV inhibitors to engineered N30C 11A19 Fab and IgG.

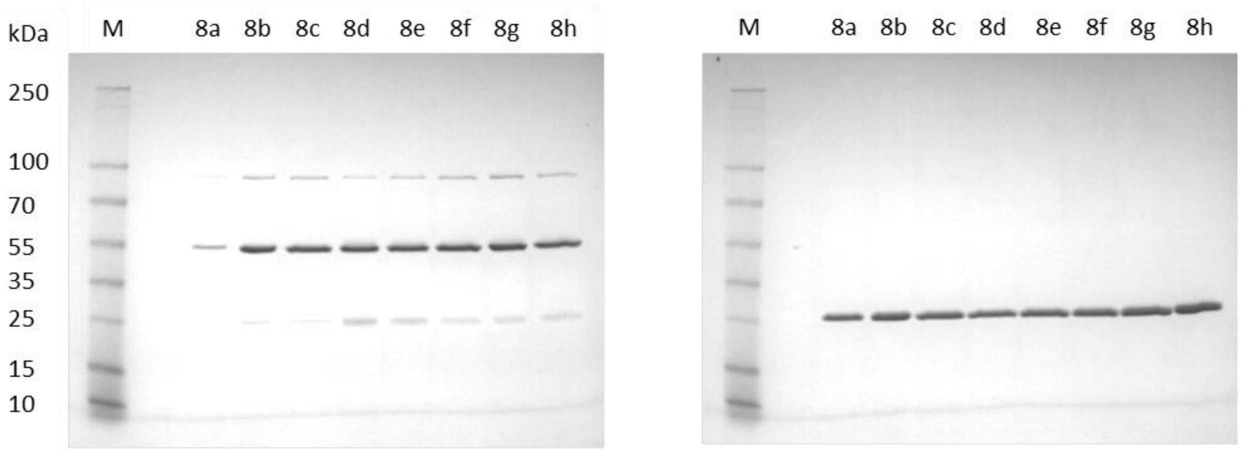

Figure S4. SDS-PAGE protein gel for Fab hybrids **8a-h** (Left: non-reducing; Right: reducing). M is the molecular weight marker (SeeBlue) used in this, as well as in all other protein gels shown. The molecular weight of each individual band in the ladder is shown in kDa. The gels are in full length with no cropping.

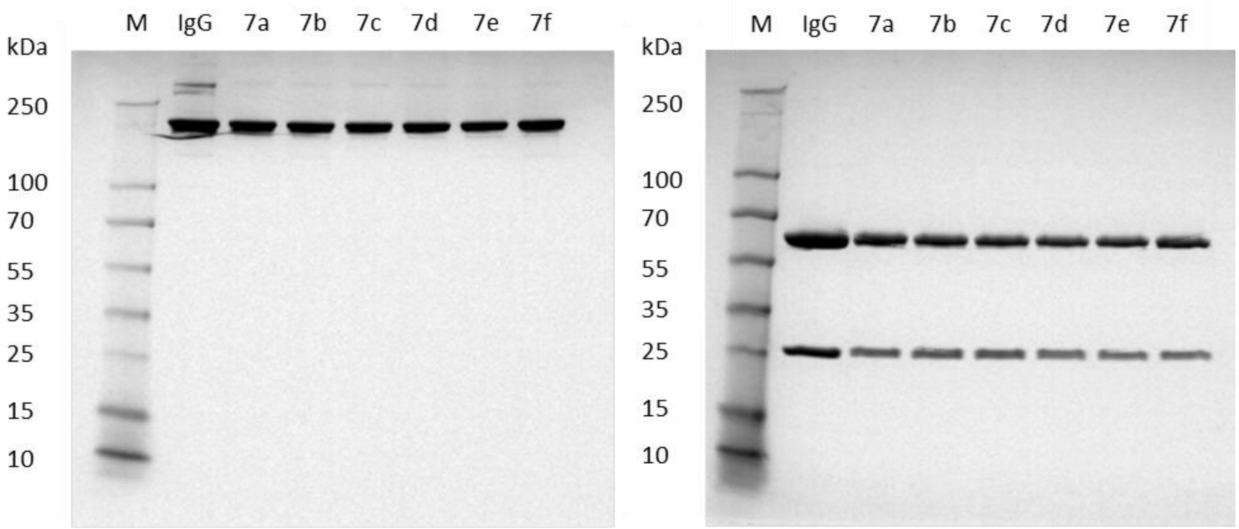

Figure S5. SDS-PAGE protein gel for IgG hybrids **7a-f** (left**,** non-reducing; right, reducing). IgG is the unconjugated cysteine engineered IgG 11A19 N30C variant. M is the molecular weight marker (SeeBlue) used in this, as well as in all other protein gels shown. The molecular weight of each individual band in the ladder is shown in kDa. The gels are in full length with no cropping.

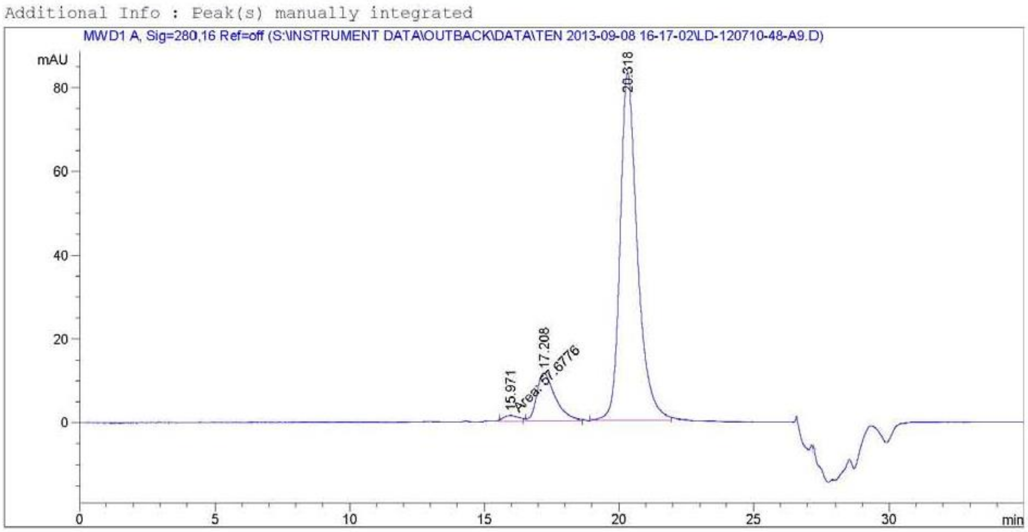

Figure S6. SEC chromatogram for Fab hybrid **8d.** The buffer composition was 50 mM Tris, 100 mM NaCl, pH 7.8. The monomer state was estimated at 86.2%.

Table S1. Binding of DPP-IV by wild-type 11A19 Fab and mutants.

	Wild-type	S28C	G67C	N30C
K_D (nM)	1.8	5.7	11.0	2.9

Table S2. Mass spectral data for acetamide controls **6a-h**.

compound	n (# of PEG units)	MS (ESI, positive ion) m/z $[M+1H]^{1+}$
**6a**	3	576.2
**6b**	4	620.3
**6c**	5	664.2
**6d**	6	708.2
**6e**	7	752.2
**6f**	8	796.5
**6g**	10	884.5
**6h**	11	928.5

Table S3. Mass spectral data for bromoacetamide reagents **5a-h**.

compound	n (# of PEG units)	MS (ESI, positive ion) m/z $[M+1H]^{1+}$
**5a**	3	576.2
**5b**	4	620.3
**5c**	5	664.2
**5d**	6	708.2
**5e**	7	752.2
**5f**	8	796.5
**5g**	10	884.5
**5h**	11	928.5

Table S4. Characterization data for Fab-hybrids.

Cmpd	n (# of PEG units)	TOF-MS crown peak (amu)	SEC %monomer (280 nm)	Concentration (mg/mL)	%Protein recovery (conjugation yield)
**8a**	n=3	47452	86.9	2.13	77
**8b**	n=4	47496	88.4	2.13	77
**8c**	n=5	47540	87.5	2.22	80
**8d**	n=6	47584	90.1	2.22	80
**8e**	n=7	47628	87.8	2.19	79
**8f**	n=8	47672	87.1	2.2	79
**8g**	n=10	47760	84.6	2.28	82
**8h**	n=11	47804	85.2	2.24	81

Table S5. Characterization data for IgG hybrids.

Cmpd	n (# of PEG units)	TOF-MS crown peak (amu)	TOF-MS LC+1	SEC %monomer (280 nm)	Concentration (mg/mL)	%Protein recovery (conjugation yield)
**7a**	n=4	147625	23605	97.9	1.99	30
**7b**	n=5	147709	23650	98	1.98	30
**7c**	n=6	147800	23693	97.3	2.55	39
**7d**	n=7	147886	23738	97.8	2.37	36
**7e**	n=8	147976	23781	98.7	2.53	38
**7f**	n=10	148154	23870	98.5	2.36	36

Table S6. Data for measured IC_{50}'s (M)

cmpd	6	7	8	9
n	$(PEG)_n$-SM	IgG-$(PEG)_n$-SM	Fab-$(PEG)_n$-SM	Fc-$(PEG)_n$-SM
3	1.41E-07		9.56E-07	7.57E-07
4	1.00E-07	2.16E-05	6.58E-07	6.26E-07
5	1.46E-07	5.95E-10	1.37E-09	4.43E-07
6	1.19E-07	6.15E-10	5.57E-10	3.11E-07
7	1.34E-07	7.22E-10	9.75E-10	2.66E-07
8	1.24E-07	7.20E-10	1.03E-09	
9				
10	1.52E-07	8.36E-10	9.25E-10	
11	1.60E-07		1.30E-09	

Table S7. X-ray crystallography data collection and refinement statistics

	Co-crystal structure of compound **7** (n=6, X=Fab)
**Data collection**	
Space group	P1
Wavelength (Å)	1.000
Cell dimensions	
a (Å)	120.32
b (Å)	123.23
c (Å)	129.02
α (°)	62.344
β (°)	77.206
γ (°)	75.914
Resolution (Å)	29.96–2.80 (2.85–2.80)
R_{mrg}	12.2 (69.0)
$I/\sigma I$	5.4 (1.0)
Completeness (%)	89.0 (72.7)
Redundancy	2.7
**Refinement**	
Resolution (Å)	2.8
Number of reflections	
Total	122463
Working set	116014
Test set	6449
R_{factor}/R_{free}	0.259/0.303
No. atoms	
Protein	33315
Ligand/ion	139
Water	188
B-factors	
Protein	56.66
Ligand/ion	61.53
Water	25.78
R.M.S. Deviations	
Bond lengths (Å)	0.012
Bond angles (°)	1.482

One crystal was used to collect dataset.
Values in parentheses are for the highest resolution shell.
$R_{factor} = \sum | F_o - F_c | / \sum F_o$, where F_o and F_c are observed and calculated structure factors, respectively, R_{free} was calculated from a randomly chosen 5% of reflections excluded from the refinement, and R_{factor} was calculated from the remaining 95% of reflections.
R.M.S.D. is the root-mean-square deviation from ideal geometry
